# Post-thalamotomy Changes Mimicking Cavernous Malformations on MRI: A Case Report of a Historical Surgical Treatment

**DOI:** 10.7759/cureus.82380

**Published:** 2025-04-16

**Authors:** Maaya Miyakoshi, Hiroki Mukai, Kaoru Yoshida, Akiyuki Uzawa, Yoshinori Higuchi, Takashi Uno

**Affiliations:** 1 Radiology, Chiba University Hospital, Chiba, JPN; 2 Neurology, Chiba University Graduate School of Medicine, Chiba, JPN; 3 Neurosurgery, Chiba University, Chiba, JPN; 4 Radiology, Chiba University Graduate School of Medicine, Chiba, JPN

**Keywords:** cervical dystonia, deep brain stimulation, post-thalamotomy changes, stereotactic neurosurgery, thalamotomy

## Abstract

The patient was a 65-year-old man with cervical dystonia onset at age six who had been treated at Chiba University Hospital. He was diagnosed and followed up with cavernous malformations and chronic cerebral infarctions based on an MRI. However, during the re-evaluation of MRI, lesions with low signal intensity (SI) cores surrounded by high SI rims were observed in bilateral thalami and left subthalamic nucleus on T2-weighted images, which differed from typical cavernous malformations. In addition, symmetrical scarring changes were noted in the frontal lobes and bilateral parietal bones, potentially corresponding to the thalamic and subthalamic nucleus lesions. Upon reviewing medical history, it was revealed that the patient had undergone thalamotomy in 1963 and 1964. The literature review suggested the use of procaine-oil blocking during thalamotomies of that era. Chemical shift imaging was added, and the presence of fat was confirmed in bilateral thalamic lesions with high SI on in-phase and low SI on out-of-phase images. Imaging findings resulting from obsolete treatments can be unfamiliar and mistaken for pathological conditions. Investigating the history of suspected treatments can lead to definitive diagnoses through imaging studies.

## Introduction

Dystonia is a movement disorder characterized by sustained or intermittent muscle contractions that result in abnormal, often repetitive movements, postures, or both [1]. Currently, dystonia is primarily treated with medication, and the efficacy of anticholinergic drugs, baclofen, benzodiazepines, and L-DOPA (levodopa) has been reported [2-3]. However, before the introduction of L-DOPA in 1960, surgical treatments were the mainstay for managing involuntary movement disorders [4]. While current surgical options include deep brain stimulation (DBS) and magnetic resonance-guided focused ultrasound (MRgFUS), earlier approaches involved various exploratory procedures, such as anterior lateral cordotomy [5], primary motor cortex resection [6], and pedunculotomy [7]. Here, we report a case of a patient who underwent thalamotomy for dystonia in the 1960s, in whom lesions initially followed up as cavernous malformations were later re-evaluated using MRI and diagnosed as post-thalamotomy changes.

## Case presentation

Patient history

A 65-year-old man with cervical dystonia onset at age six was being followed at the Department of Neurology, Chiba University Hospital. He had previously been diagnosed with cavernous malformations in bilateral thalami and left subthalamic nucleus, as well as chronic infarctions in bilateral frontal lobes. MRI was performed for follow-up purposes.

Imaging findings

**Figure 1:**
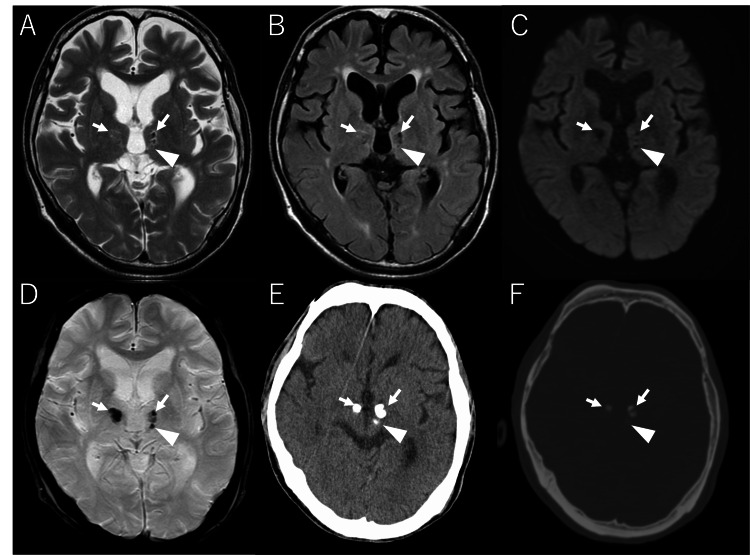
Current MR images (A-D); CT images from five years ago (E-F). Lesions are observed in the bilateral thalami (arrows) and left subthalamic nucleus (triangle). The central areas of the lesions show low signal intensity on T2WI (A), FLAIR (B), DWI (C), and T2*WI (D). On T2WI, high signal intensity is observed at the margins. CT demonstrates obvious hyperdensity (E,F). FLAIR: fluid-attenuated inversion recovery, DWI: diffusion-weighted imaging.

MRI revealed lesions with low signal intensity (SI) cores surrounded by high SI rims in bilateral thalami and left subthalamic nucleus on T2-weighted images (Figure 1). These lesions demonstrated obvious hypointensity on T2*-weighted images. Linear encephalomalacia was observed in bilateral frontal lobes. These findings remained unchanged compared to the oldest MRI available eight years prior in our picture archiving and communication system. CT images from five years ago showed calcification-like density in bilateral thalami and left subthalamic nucleus lesions. The short-axis diameter was 6 mm each for the right thalamic and left subthalamic lesions, 4 mm each for the two left thalamic lesions. On previous imaging, lesions included high signal intensity on T1-weighted imaging and showed low signal intensity on susceptibility-weighted imaging (SWI), which was interpreted as hemorrhage, leading to a suspected diagnosis of cavernous malformation.

Diagnostic assessment

**Figure 2:**
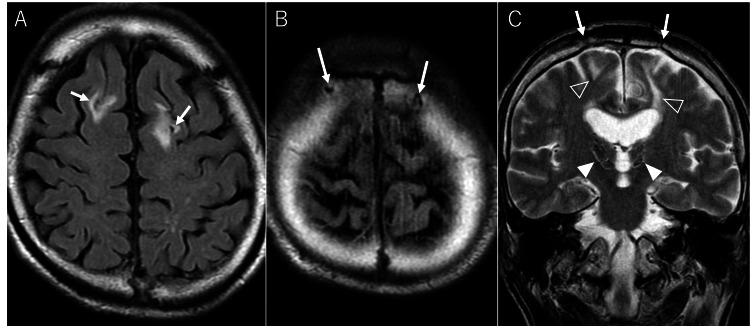
Trajectory to the thalami. FLAIR images reveal symmetrical hyperintense areas in bilateral frontal lobes (A), suggesting encephalomalacia. Above these areas, defects in bilateral parietal bones (B) like post-puncture changes were observed. On the T2WI coronal image (C), the parietal bone (arrow), frontal lobe (open arrowheads), thalamic, and subthalamic nucleus lesions (arrowheads) align linearly through the lateral ventricles. FLAIR: fluid-attenuated inversion recovery.

These lesions had been followed as cavernous malformations and chronic infarctions. However, their relatively symmetrical distribution and lack of signal heterogeneity typical of recurrent bleeding in cavernous malformations raised doubts about this diagnosis. Additionally, the current MRI showed symmetrical skull defects in the parietal bones, which appeared to be continuous with the encephalomalacia of the frontal lobe (Figure 2). Furthermore, these changes aligned linearly with the thalamic and subthalamic nucleus lesions through the lateral ventricles.

This trajectory resembled that of deep brain stimulation (DBS), suggesting treatment-related changes. Upon reviewing medical records, we found a 20-year-old note stating, "Thalamotomy was performed in July 1963 and August 1964, improving dystonia symptoms." We concluded that these imaging findings likely represented post-thalamotomy changes and investigated the relationship between this procedure and the imaging findings.

In Japan, stereotactic surgery with procaine-oil blocking, developed by Hirotaro Narabayashi, had been performed around 1950 [8]. We hypothesized that the patient's thalamotomy in 1963 and 1964 likely employed Narabayashi's stereotactic method.

**Figure 3:**
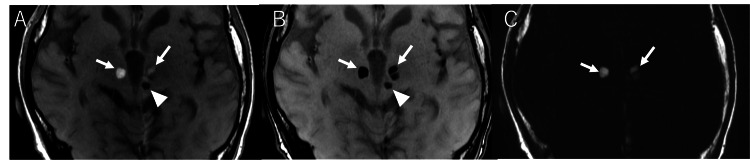
Chemical shift imaging on MRI. In chemical shift imaging using the Dixon method, the bilateral thalamic lesions show signal reduction on out-of-phase images (A: arrows) compared to in-phase images (B: arrows), whereas the left cerebral peduncle lesion does not show signal reduction (A and B: arrowheads). Fat images demonstrate high signal intensity in the bilateral thalamic lesions (C: arrows).

Narabayashi's thalamotomy with procaine-oil blocking technique involved skull trepanation, advancing a needle to the target coordinates and injecting procaine hydrochloride oil suspension [9]. To confirm that stereotactic surgery was performed in this case, we sought to demonstrate remnants of the procaine hydrochloride oil suspension, specifically fat components, in the bilateral thalamic and left subthalamic nucleus lesions. Given that the findings were considered non-urgent, chemical shift imaging was added during the subsequent follow-up MRI performed three years later. The bilateral thalamic lesions showed high SI on in-phase and low SI on out-of-phase images, confirming fat content (Figure 3). However, the left subthalamic nucleus lesion demonstrated low SI on both in-phase and out-of-phase images, indicating no fat content.

In Narabayashi's thalamotomy procedure with procaine-oil blocking, an aqueous procaine hydrochloride solution was initially injected to confirm effectiveness, followed by a procaine hydrochloride oil suspension for sustained effect [9]. The difference in fat content suggests that the aqueous procaine hydrochloride solution was first injected into the left subthalamic nucleus without the desired effect, then redirected to the thalamus, where, upon confirmation of efficacy, a procaine hydrochloride oil suspension was subsequently injected.

## Discussion

Various experimental surgical treatments for movement disorders were carried out between the late 1900s and the 1930s, with a rudimentary understanding of the pathogenesis and neuroanatomical basis for motor control [4]. These included anterior lateral cordotomy to interrupt proprioceptive input [5], primary motor cortex resection to interrupt the pyramidal tract [6], and pedunculotomy to interrupt extrapyramidal pathways [7]. However, these procedures were highly invasive, often resulting in mortality or severe postoperative motor deficits. Subsequently, less invasive approaches were pursued, leading to the development of stereotactic neurosurgery by Spiegel and Wycis in 1947 [10].

In Japan, Hirotaro Narabayashi began performing pallidotomy for Parkinson's disease around 1950 [8]. Hassler et al. confirmed the efficacy of thalamotomy in 1960 [11]. With the development of L-DOPA therapy in the 1960s, surgical treatments declined. However, as long-term side effects of L-DOPA became apparent, surgical interventions regained attention. In 1987, Benabid et al. invented DBS [12]. DBS, being less invasive and reversible, largely replaced ablative procedures. Recently, MR-guided focused ultrasound (MRgFUS) has emerged as a non-invasive stereotactic option [13].

Our patient underwent thalamotomy in 1963 and 1964, during the period when surgical treatments were predominant. Typically, thalamotomy was performed unilaterally due to severe side effects such as gait disturbance and cognitive impairment associated with bilateral procedures [9]. In this case, post-thalamotomy changes were observed bilaterally in the thalami. The two separate procedures in 1963 and 1964 likely represent staged bilateral thalamotomies to mitigate side effects.

Chemical shift imaging confirmed fat content in the lesions. Narabayashi's pallidotomy technique involved mixing contrast agents with procaine hydrochloride solution/oil suspension for intraoperative position correction using plain radiographs. The high-density appearance of bilateral thalamic and left subthalamic nucleus lesions on CT, initially assumed to be calcifications in cavernous malformations, might reflect residual contrast agents. However, various substances were experimented with as contrast agents at the time, making it difficult to determine the exact agent used. The obvious hypointensity on T2*WI can also indicate dystrophic calcification or hemosiderin deposition post-surgery. An inherent limitation is the impossibility of obtaining a histopathological diagnosis.

By 1965, approximately 25,000 surgical procedures had been performed to address movement disorders [14]. Over time, cases treated with outdated surgical techniques have become increasingly uncommon. However, cavernous malformations, which were suspected in the current case, are estimated to occur in 0.4-0.8% of the general population and are often discovered incidentally in routine clinical practice [15]. Differences in clinical exposure to such cases may contribute to diagnostic inaccuracies.

While DBS is commonly performed today, the presence of implanted electrodes makes misdiagnosis unlikely. Although less frequent than DBS, other procedures such as radiofrequency ablation (RFA), stereotactic radiosurgery (SRS), and, more recently, MRgFUS are also being utilized. In RFA, the ablation site may mimic findings typically seen in infarction or post-hemorrhagic changes. Therefore, it is crucial to identify the puncture tract. In contrast, because SRS and MRgFUS are non-invasive, they do not leave traces outside the targeted area. When no puncture tract is visible, postoperative changes should be suspected based on the imaging findings' location, and a detailed patient history should be obtained.

While these neurosurgical interventions are primarily utilized in the management of Parkinson’s disease, their application in dystonia remains relatively limited [16]. Apart from DBS, other modalities, such as radiofrequency ablation (RFA), stereotactic radiosurgery (SRS), and MRgFUS, are less commonly employed, making their postoperative imaging findings unfamiliar to clinicians not practicing in highly specialized centers. Consequently, a thorough understanding of the disease-specific indications for each technique is crucial for the accurate interpretation of neuroimaging and its clinical correlation.

Imaging findings initially misdiagnosed as benign lesions are often followed over time without histopathological confirmation, as in the present case. Therefore, it is important to consider the possibility of post-treatment changes based on imaging, review the patient's medical history, and refer to previous literature. When appropriate, additional imaging studies can contribute to achieving a more accurate diagnosis.

## Conclusions

In this case, imaging findings that initially appeared atypical for cavernous malformations prompted a reevaluation based on historical treatment methods, leading to the identification of post-thalamotomy changes. Many early surgical treatments are now unfamiliar, and their residual effects can sometimes be mistaken for pathological conditions. Such misinterpretations may cause unnecessary anxiety for patients and lead to unwarranted diagnostic tests and follow-up evaluations. When encountering findings that known diseases cannot readily explain, it is essential to consider alternative possibilities, including post-treatment changes. Given the limited literature on imaging findings of surgical procedures that are no longer performed, this case report provides valuable insight.
